# Presepsin as a promising biomarker for early detection of post-operative infection in children

**DOI:** 10.3389/fped.2023.1036993

**Published:** 2023-03-13

**Authors:** Niken Wahyu Puspaningtyas, Mulya Rahma Karyanti, Tiara Nien Paramita, Hikari Ambara Sjakti, Nina Dwi Putri, Bambang Tridjaja, Piprim Basarah Yanuarso, Kshetra Rinaldhy, Ahmad Yani, Pramita Gayatri

**Affiliations:** ^1^ Department of Pediatrics, Cipto Mangunkusumo National Central Hospital, Faculty of Medicine, Universitas Indonesia, Jakarta, Indonesia; ^2^ Department of Surgery, Cipto Mangunkusumo National Central Hospital, Faculty of Medicine, Universitas Indonesia, Jakarta, Indonesia

**Keywords:** children, diagnostic study, infection, presepsin, surgery

## Abstract

**Background:**

Post-operative systemic inflammation response syndrome (SIRS) is an event that results from surgical trauma, white blood cells contact activation, and intra-surgical bacterial translocation, which is difficult to distinguish from sepsis. Presepsin is a novel biomarker that is increased since the early stages of bacterial infection and can be used to confirm the diagnosis of post-operative infectious complications. This study aimed to investigate the diagnostic performance of presepsin for post-operative infectious complications compared to other well-known biomarkers.

**Method:**

This cross-sectional study included 100 post-operative patients admitted to Cipto Mangunkusumo National Hospital and Bunda Hospital in Jakarta, Indonesia. The objective was to identify the optimal cutoff and trend of plasma presepsin concentration on the first and third day after surgery and to compare them with other biomarkers.

**Result:**

Plasma presepsin level was higher in the infection group compared to the non-infection group (median 806.5 pg/ml vs. 717 pg/ml and 980 pg/ml vs. 516 pg/ml on the first and third day, respectively). Presepsin levels tended to increase on the third post-operative day (median + 252 pg/ml) in children with infection. The opposite trend was observed in the non-infection group from the first to the third day (median -222.5 pg/ml). Presepsin delta, a three-day difference between the first and third post-operative day, had the best diagnostic performance compared to other biomarkers (Area Under the Curve 0.825). The optimal cutoff for presepsin delta to diagnose post-operative infection was +90.5 pg/ml.

**Conclusion:**

Serial assessments of presepsin levels on the first and third days post-surgery and their trends are helpful diagnostic markers for clinicians to detect post-operative infectious complications in children.

## 1. Introduction

Post-operative systemic inflammation response syndrome is caused by surgical trauma, white blood cells (WBCs) contact activation, and intra-surgical bacterial translocation. This condition is frequently confused with sepsis (1). Microbiological culture is the gold standard to diagnose post-operative infection with high specificity; however, it has low sensitivity and a high potential for contamination (2). Culture requires a long time to develop, which is not ideal for early detection of infection. Biological infection markers play a significant role in early detection of post-operative infections. The most commonly studied biological markers are C-reactive protein (CRP) and procalcitonin (PCT), but both have limitations in differentiating infection from non-infection inflammatory responses after surgery (3–5).

In the past decade, presepsin (sCD14-ST) has been studied as a novel marker of infection. It is a biological immune marker with a potential role in detecting infection, especially in its early stages (6–8). Plasma presepsin levels start to increase 2 h after the onset of infection (9). Several studies within the past decade have shown presepsin as a promising biological marker to diagnose sepsis in adults (10–12). Compared to other markers such as PCT and CRP, it is more specific for bacterial infection due to its direct involvement in the pathomechanism of infection and is a fragment of the CD14 receptor-lipopolysaccharide complex (8).

At the time of this study, only one systematic review and meta-analysis had been published regarding the diagnostic performance of presepsin in pediatric sepsis. It reported better performance of presepsin (Area Under the Curve/AUC 0.94) compared to CRP (0.51) and PCT (0.76) in detecting sepsis in children (10). There have been no published studies explicitly reporting the role of presepsin in post-operative infection in children. Presepsin is also not readily accessible in Indonesia and is seen as an expensive substitute for other biomarkers. This study aimed to evaluate the diagnostic performance of presepsin in detecting post-operative infection in children and compare it with microbiological culture as the gold standard.

## 2. Method

### 2.1. Patient enrollment

This cross-sectional study was conducted on children aged 1 month-18 years in Cipto Mangunkusumo National Hospital and Bunda Hospital in Jakarta, Indonesia. The Ethics Committee of the Faculty of Medicine, Universitas Indonesia, approved this study. Samples were collected prospectively through consecutive sampling from January to November 2021. Sample size was estimated by using the sample size calculation formula for diagnostic test (13). The inclusion criteria were post-operative children aged 1 month to 18 years with parental consent. However, children with acute kidney injury, chronic kidney disease with decreased glomerular filtration rate, or pre-operative infection were excluded. The outcome was a post-operative infection, defined as infection occurring within 30 or 90 days (for specific procedures involving implants) after surgery and proven by culture.

### 2.2. Sample collection

Complete blood count, plasma presepsin and procalcitonin levels, and blood culture samples were collected within 24 h after surgery. The second presepsin and procalcitonin sample was collected on the third day after surgery. Other microbiological cultures were also obtained once there were signs of infection.

Complete blood count was performed using Sysmex XT 2000i^TM^ with hydrodynamic focusing method, flow cytometry method using a semiconductor laser, and sodium lauryl sulfate-hemoglobin method. Presepsin was measured using the PATHFAST^TM^ immunoanalyzer (Mitsubishi Chemical Medience Corporation, Tokyo, Japan). The blood culture was analyzed using BACTEC^TM^ with BacT/Alert method (Biomeriux, France).

### 2.3. Statistical analysis

Data analysis was conducted with the 26th version of the Statistical Package for the Social Sciences (SPSS) statistical program. Receiver operating characteristic (ROC) analysis was used to determine the presepsin AUC and cutoff value to diagnose post-operative infection, and the optimal cutoff value was chosen using Youden's index. Furthermore, the sensitivity, specificity, negative predictive value (NPV), positive predictive value (PPV), and likelihood ratio (LR) were calculated following the ROC curves for each biomarker. Statistical significance was set at *p* < 0.05.

## 3. Results

### 3.1. Patient characteristics

One hundred post-operative subjects as summarized in Table 1 with a median age of 7 years and 5 months were recruited. Most subjects were female (54%) and aged 5 years and above (52%). Six patients had non-infectious post-operative complications.

**Table 1:** Subject characteristics.

Variable	Total	Post-operative Infection		*p*
		Yes (*n* = 18)	No (*n* = 82)	
**Sex**, *n* (%)				
Male	46 (46.0)	7 (38.8)	39 (47.5)	0.504
Female	54 (54.0)	11 (61.1)	43 (52.5)	
**Age,** *n* (%)				
Infant (1 month—1 year)	17 (17.0)	8 (44.4)	9 (11.0)	0.003
Toddler (1–5 years)	31 (31.0)	4 (22.2)	27 (32.9)	
Child (> 5 years)	52 (52.0)	6 (33.3)	46 (56.1)	
**Nutritional Status,** *n* (%)				
Well-nourished	51 (51.0)	11 (61.1)	40 (48.8)	0.123
Moderately malnourished	26 (26.0)	2 (11.1)	24 (29.3)	
Severely malnourished	14 (14.0)	5 (27.8)	9 (11.0)	
Overweight	1 (1.0)	0 (0.0)	1 (1.2)	
Obese	8 (8.0)	0 (0.0)	8 (9.8)	
**ASA Class**, *n* (%)				
I	10 (10.0)	1 (5.6)	9 (11.0)	0.155
II	42 (42.0)	4 (22.2)	38 (46.3)	
III	45 (45.0)	12 (66.7)	33 (40.2)	
IV	3 (3.0)	1 (5.6)	2 (2.4)	
**Comorbidity,** *n* (%)				
Yes	54 (54.0)	10 (55.6)	44 (53.7)	0.884
No	46 (46.0)	8 (44.4)	38 (46.3)	
**Type of Surgery**, *n* (%)				
Emergency	14 (14.0)	6 (33.3)	8 (9.8)	0.018*
Elective	86 (86.0)	12 (66.7)	74 (90.2)	
**Surgery location,** *n* (%)				
Abdomen	35 (35.0)	11 (61.1)	24 (29.3)	0.130
Nervous system	15 (15.0)	3 (16.7)	12 (14.6)	
Head, ears, nose, and throat	14 (14.0)	1 (5.6)	13 (15.9)	
Heart	11 (11.0)	0 (0.0)	11 (13.4)	
Bone	9 (9.0)	0 (0.0)	9 (11.0)	
Kidney and urinary system	7 (7.0)	0 (0.0)	7 (8.5)	
Lungs	2 (2.0)	1 (5.6)	1 (1.2)	
Reproductive system	2 (2.0)	1 (5.6)	1 (1.2)	
Multiorgan	2 (2.0)	1 (5.6)	1 (1.2)	
Vascular	1 (1.0)	0 (0.0)	1 (1.2)	
Extremities	1 (1.0)	0 (0.0)	1 (1.2)	
**Type of Surgical Wound**, *n* (%)				
Clean	45 (45.0)	6 (33.3)	39 (47.6)	0.053
Clean-contaminated	45 (45.0)	8 (44.5)	37 (45.1)	
Contaminated	3 (3.0)	2 (11.1)	1 (1.2)	
Dirty	7 (7.0)	2 (11.1)	5 (6.1)	
**Duration of Surgery,** *n* (%)				
≤ 2 h	9 (9.0)	1 (5.6)	8 (9.8)	1.000*
>2 h	91 (91.0)	17 (94.4)	74 (90.2)	
**Use of Prophylaxis Antibiotic**, *n* (%)				
Yes	82 (82.0)	14 (77.8)	68 (82.9)	0.480
No	3 (3.0)	0 (0.0)	3 (3.7)	
Therapeutic antibiotic	15 (15.0)	4 (22.2)	11 (13.4)	
**Duration of Central Vein Catheter (Days),** *n* (%)				
**1-7** days	57 (57.0)	3 (16.7)	54 (65.9)	<0.001
> 7 days	28 (28.0)	15 (83.3)	13 (15.9)	
No catheter	15 (15.0)	0 (0.0)	15 (18.3)	
**Duration of Urinary Catheter (Days),** *n* (%)				
< 3 days	77 (77.0)	9 (50.0)	68 (82.9)	<0.001
> 3 days	16 (16.0)	9 (50.0)	7 (8.5)	
No catheter	7 (7.0)	0 (0.0)	7 (8.5)	
**Duration of Ventilator Use (Days),** *n* (%)				
1-7 days	74 (74.0)	16 (88.9)	58 (70.7)	0.144*
> 7 days	26 (26.0)	2 (11.1)	8 (9.8)	
**Post-operative Hyperglycemia**				
Yes	12 (13.2)	2 (11.1)	10 (13.7)	1.000*
No	79 (86.8)	16 (88.9)	63 (86.3)	
**Outcome**				
Survived	99 (99.0)	17 (94.4)	83 (100)	0.180*
Death	1 (1.0)	1 (5.6)	0 (0.0)	
**Non-infectious Complication**				
Yes	6 (6.0)	3 (16.7)	3 (3.6)	<0.001
No	94 (94.0)	15 (83.3)	79 (96.4)	

* Fisher's exact test.

The prevalence of post-operative infection was 18 of 100 subjects (18%). One-third of the infection (*n* = 5) occurred in multiple organs. Twenty-seven bacteria and 2 fungi were cultured from 18 patients. Most infections were caused by Gram-negative bacteria, such as *Escherichia coli* and *Klebsiella pneumoniae* with 8 and 7 cultures, respectively.

### 3.2. Diagnostic performance of presepsin and comparison with other acute phase reactants

The difference between the first and third-day presepsin levels was calculated as “delta presepsin.” This variable was determined to evaluate the trend of presepsin levels on the first and third day after surgery. Table 2 shows the median presepsin levels on the first and third day after surgery and delta presepsin levels. In the non-infection group, presepsin levels decreased on the third day but the opposite was observed in the infection group. There was a significant difference in the third-day presepsin and delta presepsin levels in the infection group compared to the non-infection group. Significant differences in the third-day presepsin, first- and third-day PCT were observed between the infection and non-infection groups (*p* < 0.05).

**Table 2:** Median comparison of biomarkers of infection and non-infection group.

Variable	Post-operative Infection		*p*
	Yes	No	
**Presepsin, median (IQR)**			
First day	806.5 (318.5–1288.5)	717 (467.25–1306.75)	0.414
Third day	980 (716.25–1612)	516 (307.5–878.25)	**< 0** **.** **001**
Delta	252 (127.75–559.5)	−222.5 ((-652)–(-21.0))	**< 0** **.** **001**
**Procalcitonin, median (IQR)**			
First day	1.19 (0.48–4.98)	0.19 (0.10–0.75)	**< 0** **.** **001**
Third day	2.48 (0.57–10.31)	0.59 (0.16–1.87)	**0** **.** **004**
**WBCs, median (IQR)**	10,450 (7598–15,045)	13,695 (10,580–17,908)	0.050

Mann Whitney test; IQR: interquartile range.

**Figure 1:**
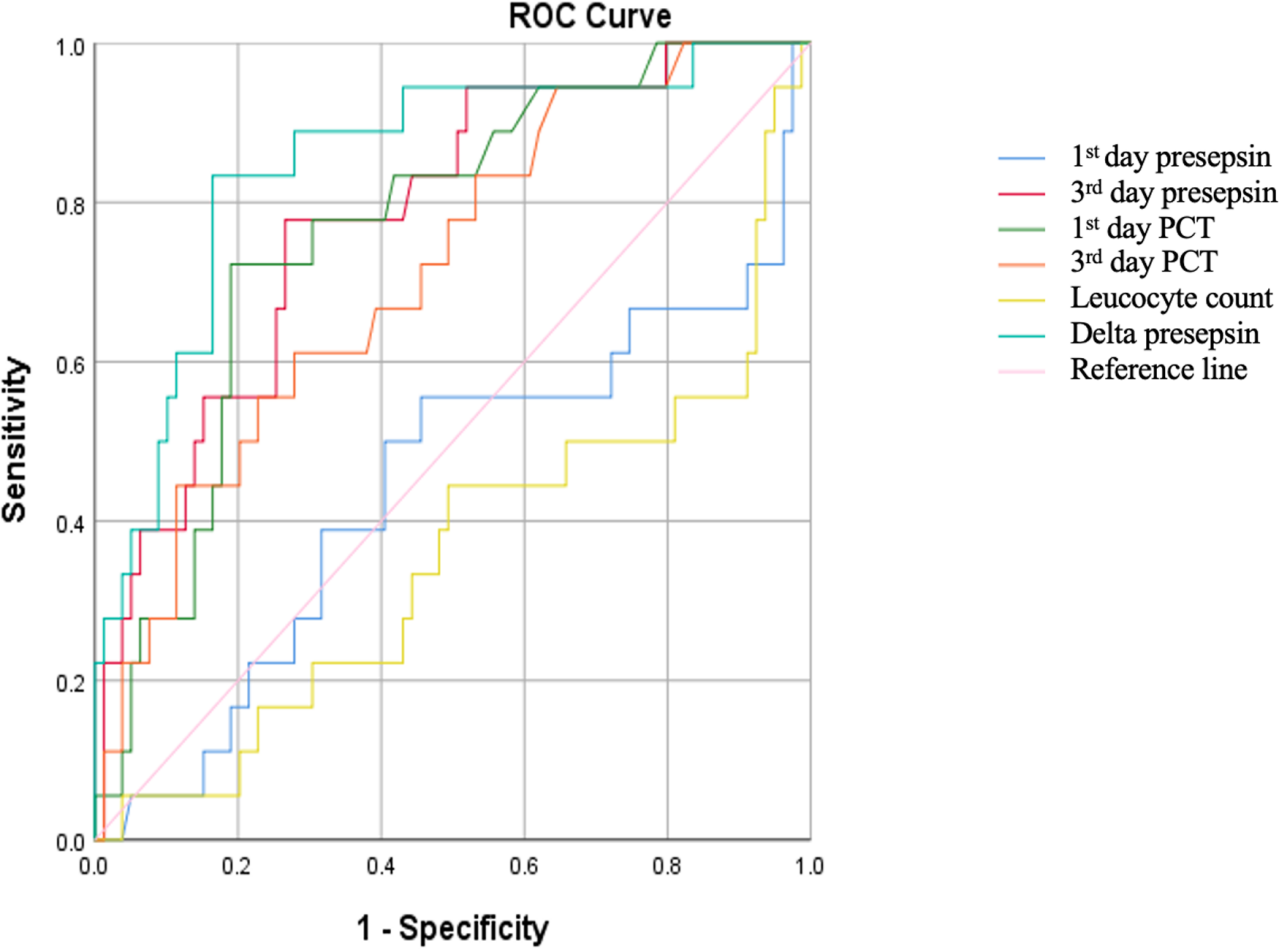
ROC curve of presepsin and other biomarkers.

Figure 1 shows the receiver operating characteristic analysis of the first- and third-day presepsin levels, delta presepsin, and the first- and third-day PCT levels. Further analysis was performed to evaluate the optimal cutoff for variables with AUC > 0.5, namely third-day presepsin, first-day PCT, third-day PCT, and delta presepsin.

The ROC curve showed optimal cutoff for third-day presepsin, delta presepsin, first-day PCT, and third-day PCT were 761 pg/ml, + 90.5 pg/ml, 0.73 mg/L, and 1.085 mg/L, respectively. Table 3 exhibits the diagnostic performance of each biomarker. Delta presepsin had the highest AUC and overall diagnostic performance compared to other biomarkers. There was no statistical difference between the AUC values of the compared biomarkers (*p* = 0.418).

**Table 3:** Diagnostic performance of presepsin and other biomarkers.

Parameter	Third-Day Presepsin (761 pg/ml)	Delta Presepsin (+90.5 pg/ml)	First-Day PCT (0.73 mg/l)	Third-Day PCT (1.085 mg/l)
**Sensitivity (95% CI)**	77.78 (52.36–93.59)	83.53 (58.58–96.42)	72.22 (46.52–90.31)	66.67 (40.99–86.66)
**Specificity (95% CI)**	71.95 (60.94–81.32)	84.15 (74.42–91.28)	75.0 (64.06–84.01)	61.26 (49.70–71.94)
**Positive Predictive Value (95% CI)**	37.84 (28.45–48.23)	53.57 (40.21–66.44)	39.39 (28.78–51.12)	27.91 (20.16–37.24)
**Negative Predictive Value (95% CI)**	93.65 (86.01–97.25)	95.83 (89.07–98.48)	92.31 (84.93–96.23)	89.09 (80.59–94.14)
**Positive Likelihood Ratio (95% CI)**	2.77 (1.81–4.24)	5.26 (3.06–9.02)	2.89 (1.80–4.65)	1.72 (1.12–2.64)
**Negative Likelihood Ratio (95% CI)**	0.31 (0.13–0.74)	0.20 (0.07–0.56)	0.37 (0.17–0.79)	0.54 (0.28–1.07)
**Accuracy (95% CI)**	73 (63.20–81.39)	84.0 (75.32–90.57)	74.49 (64.69–82.76)	62.24 (51.88–71.84)
**AUC (95% CI)**	**0.772** (**0.658**–**0.887)**	**0.853** (**0.751**–**0.955)**	**0.767** (**0.651**–**0.881)**	**0.717** (**0.592**–**0.843)**

## 4. Discussion

In this study, post-operative infection was prevalent in 18% of patients. Specific data on children remain scarce, and most are focused on surgical site infection (SSI). These findings are comparatively higher than those of other studies (14–16), which could be attributed to the differences in the population. The World Health Organization reported that the prevalence of hospital-acquired infections in low-middle income countries is at least 2–3 times higher than that in high-income countries (17).

The median first-day and third-day presepsin levels in this the current study were 725 pg/ml and 531.5 pg/ml, respectively. This result was higher than the average presepsin level in healthy adult subjects, i.e., 89–382 pg/ml (18). Studies have reported increased plasma presepsin levels after significant surgeries, possibly related to the inflammatory response to surgical trauma (19). Surgical incision disturbs the skin barrier, causing pathogenic bacterial translocation from the skin and from outside to the inside of the body. This may induce pathogen-associated molecular patterns (PAMP) release, which induces sCD14 or presepsin production (18–20).

The median post-operative presepsin levels on the first and third days in the non-infection group, respectively, were 717 and 516 pg/ml. This result was interesting as the non-infection group results were similar to the median presepsin level to detect sepsis in other studies. A systematic review by Yoon et al. reported a 650 pg/ml cutoff with 0.983 AUC to detect sepsis in children (10). Higher post-operative presepsin levels indicate that many bacterial translocations occur intraoperatively. Therefore, the usual presepsin cutoff for sepsis cannot be used to detect post-operative infection.

Generally, presepsin levels decreased from the first to the third post-operative day. The study by Koakutsu et al. on 118 post-spinal surgery subjects reported presepsin increased soon after surgery and dropped back to the preoperative level at 1 week after surgery (21). The median delta presepsin in the non-infection group was −222.5 pg/ml. A study by Takeuchi et al*.* on post-esophagostomy surgery subjects reported a median first- and third-day post-operative presepsin levels of 313 pg/ml (IQR/interquartile range 218–398) and 276 pg/ml (IQR 229–361), respectively, in the non-infection group (22). Other studies in post-cardiac surgery adult patients reported a respective first-day and third day post-operative presepsin levels of 568 pg/ml (IQR 363–858) and 422 pg/ml (IQR 293–619) in the non-infection group (23).

This study showed a significant difference between presepsin levels on the third post-operative day and delta presepsin levels in the infected and non-infected groups. The results showed that the first-day post-operative presepsin level did not differ significantly between the infection and non-infection groups. This is similar to other findings regarding perioperative presepsin level stating that the fluctuation of perioperative presepsin level can be a predictor for post-operative complication (19, 23–27). Popov et al. reported subjects with a persistent increase of presepsin level until the sixth day after surgery had a significantly higher risk of complications (23). Another *al*. revealed that the presepsin trend in the infection group tended to increase from pre-surgery until the seventh day (22). The highest diagnostic performance of presepsin level for post-operative infection was on the fifth and seventh day following surgery. This study, however, only evaluated presepsin levels on the first and third days after surgery, and therefore the results may be less indicative of infections occurring on the third post-operative day.

Currently, PCT and WBC counts are clinicians' most commonly-used infection markers. The result of the current study revealed that presepsin trend from the first to the third post-operative day has the highest diagnostic performance in detecting post-operative infection compared to PCT or WBC count. This finding is similar to those of previously published studies. Yao et al. studied 105 post-hepatobiliary surgery subjects and reported that presepsin had the highest AUC in detecting post-operative infection compared to PCT (0.723 AUC), CRP (0.8 AUC), and neutrophil-lymphocyte ratio (0.804 AUC) (27). According to Takeuchi et al., the AUC of the fifth and seventh day post-surgery level of presepsin was higher than that of the other three biomarkers including PCT, CRP, and WBC count (22). This could be attributed to a more bacteria-specific presepsin production mechanism compared to other biomarkers, which were produced by the release of proinflammatory cytokines (20).

In this study, first- and third-day PCT level were significantly increased in the post-operative infection group. However, it was notable that PCT increased similarly from first to third day post-surgery in both infection and non-infection groups. This is in contrast with presepsin, which predictably decreased on the third day post-surgery in the non-infection group, and increased on the third day post-surgery in the infection group. The cut-off levels were also considerably lower compared to other studies (0.73 mg/L and 1.085 mg/l on the first and third day, respectively). Farias et al. reported 12.9 ng/ml cut-off in the infection group compared to 5.6 ng/ml in the non-infection group on the first day post-cardiac surgery; and 15 ng/ml (infection group) compared to 3.6 ng/ml (non-infection group) on the third day post-cardiac surgery (28). The difference might be attributed to various type of surgeries in our study, which could lead to varying degrees of post-operative inflammation.

This study showed that PCT was an effective parameter for infection especially within the first 24 h. This is highly beneficial in daily practice, as it is crucial to determine infection as soon as possible in order to start the appropriate antibiotic therapy. However, empirical antibiotic therapy was started on the first post-operative day in our samples, as all of the subjects underwent major surgeries. In such cases, presepsin may help clinicians not only to evaluate the possibility of infections; but also the response to the current antibiotic therapy. The alternate presepsin level will help clinicians to differentiate infection from clinical features of SIRS in post-operative patients. Increased presepsin levels on the third post-operative day help clinician to confirm the presence of infection and promptly implement the right course of action, such as changing the antibiotics, or increasing the dose to a dose appropriate for severe infection.

This study is the first to document post-operative presepsin levels and changes in children within the first 72 h following surgery. Furthermore, subjects with a lower preoperative glomerular filtration rate were excluded because this condition could increase plasma presepsin levels through decreased excretion (19, 20).

A limitation of this study lies in the fact that only presepsin levels on the first and third days after surgery were evaluated. The operational definition of post-operative infection is an infection occurring within 30 days post-surgery or 90 days for several specific procedures or implants (29). There were three patients with the infection on the seventh, eighth, and fourteenth day after surgery which had decreased levels of presepsin on the third day after surgery. This could be attributed to the dynamic fluctuation of presepsin levels towards the bacterial load inside the human body. The initial time of infection in these three subjects was presumed to occur after the third post-operative day but was still under the operational definition of post-operative infection. Other studies on perioperative presepsin have commonly evaluated serial presepsin levels until 14 days following surgery. Future studies can determine a more extended period of post-operative presepsin levels, ideally until the fourteenth post-operative day.

This study evaluated blood cultures on the first post-operative day. Another limitation was that the next cultural examination was conducted based on the specialist's consideration, which may vary for each subject. We also did not collect plasma presepsin and PCT along with the culture examination beyond the third post-operative day. Ideally, the plasma presepsin and PCT should be taken simultaneously together with every culture samples.

## 5. Conclusion

Both presepsin and PCT can be used as diagnostic markers in predicting post-operative infection in children. Delta presepsin (difference between first- and third-day presepsin levels) had the best diagnostic performance compared to other biomarkers.

## Data Availability

The raw data supporting the conclusions of this article will be made available by the authors, without undue reservation.
